# Midlife plasma proteomic profiles indicate altered amyloid and tau processing in former elite rugby players

**DOI:** 10.1136/jnnp-2025-336593

**Published:** 2025-10-05

**Authors:** Neil Graham, Karl Zimmerman, Jessica Hain, Erin Rooney, Ying Lee, Martina del Giovane, Thomas Parker, Mathew Wilson, Maneesh Patel, Elena Veleva, Owen Swann, Amanda J Heslegrave, Lucia M Li, Henrik Zetterberg, Daniel Friedland, Richard Sylvester, David Sharp

**Affiliations:** 1 Brain Sciences, Imperial College London, London, UK; 2 UK Dementia Research Institute Centre for Care Research and Technology, Imperial College London, London, UK; 3 Institute of Sport, Exercise and Health, UCL, London, England, UK; 4 Department of Stroke and Neuroscience, Imperial College London NHS Healthcare Trust, London, UK; 5 UK Dementia Research Institute, UCL, London, UK; 6 Department of Neurodegenerative Disease, UCL Queen Square Institute of Neurology, London, UK; 7 Acute Stroke and Brain Injury Unit, National Hospital for Neurology and Neurosurgery, London, London, UK

**Keywords:** DEMENTIA, TRAUMATIC BRAIN INJURY, HEAD INJURY, NEUROBIOLOGY

## Abstract

**Background:**

Contact sports, including rugby union, are associated with higher rates of neurodegenerative dementia, due to various underlying pathologies such as Alzheimer’s disease (AD) and chronic traumatic encephalopathy (CTE). New ultrasensitive multiplexed immunoassays may clarify disease mechanisms after repetitive head impacts (RHI) and traumatic brain injury, potentially aiding risk-stratification, early diagnosis and dementia treatment.

**Methods:**

Midlife participants in the ABHC cohort underwent plasma biomarker quantification (NULISA - NUcleic acid Linked Immuno-Sandwich Assay; n=124 markers), 3T MRI, trauma exposure ascertainment and phenotyping. Regressions quantified exposure-specific protein expression, relationship to trauma (including position) and brain atrophy, using cluster analysis to test correlates of traumatic encephalopathy syndrome (TES).

**Results:**

197 former elite rugby players and 33 controls were assessed. 24 (12.2%) met criteria for TES but none had dementia. Ex-players returned reduced plasma glial fibrillary acidic protein (GFAP), kallikrein-6 (KLK6) and synaptosomal-associated protein 25 (SNAP25). Ex-forwards specifically showed reduced plasma beta-site amyloid precursor protein cleaving enzyme 1 (BACE1), amyloid beta-38 (Aβ38), and increased phospho-tau_181_ (p-tau_181_). KLK6 was lower in ex-backs than controls. No biomarkers related to career duration, concussion load or regional brain volume, nor did clustering relate to TES.

**Conclusions:**

Ex-players showed distinctive plasma biomarker changes, more prominently in ex-forwards, possibly reflecting greater RHI exposure. Plasma KLK6, an endothelial serine protease, was reduced across the ex-player group, with potential diagnostic or prognostic utility in future. Reduced GFAP and SNAP25 in ex-forwards has an uncertain basis, while elevated p-tau-_181_ more so than p-tau_217_ points towards non-AD tau pathology. Our findings motivate longitudinal characterisation, including comparison with other neurodegenerative diseases.

## Introduction

 Rugby union participation is associated with increased rates of neurodegenerative dementia. This is ~2.7 times more common in former elite male ex-rugby players^1^, attributed to repetitive head impacts (RHI) and traumatic brain injury (TBI) exposure. However, midlife correlates of participation are poorly understood, and we lack reliable tools to stratify future dementia risk. The UK Advanced Brain Health Clinic cohort study was established to assist early dementia diagnosis, improve prognostication, risk reduction and help develop treatments.^2^

Neurodegenerative pathologies have been reported in post-mortem series of former rugby players with chronic traumatic encephalopathy (CTE), a trauma-related tauopathy, present in a number of individuals.^3^,^5^ However, a wider range of neuropathologies is described in ex-players, including Alzheimer’s disease (AD), cerebral amyloid angiopathy, TAR DNA binding protein 43 (TDP-43), cerebrovascular disease, corticobasal degeneration (CBD) and age-related tau astrogliopathy,^6^ and combinations of pathologies are common.^7^ In a large 2023 case series,^8^ comprising 31 mainly amateur ex-players, CTE was present in 68% of cases and was associated with longer career duration but not TBI history.

We have previously reported raised plasma concentrations of AD marker phospho-tau_217_ in former elite rugby players.^9^ The range of pathologies after RHI/TBI motivates measuring a broader range of proteins implicated in neurodegeneration. A recently developed multiplex panel (n=120 nervous system proteins) using NUcleic acid Linked Immuno-Sandwich Assay (NULISA, Alamar) to provide attomolar sensitivity may provide useful mechanistic insights^10^ and has revealed widespread proteomic changes after acute TBI^11^ and in AD.^12 13^

Here, we aimed to describe the high-dimensional plasma proteomic correlates of prior elite rugby participation. We hypothesised that: (i) ex-rugby players would show differential expression of plasma neurodegeneration biomarkers vs controls; (ii) these differences would relate to player position (forwards being more exposed than backs);^14 15^ these would have longer career durations and TBI exposure; (iv) differentially expressed protein concentrations would be associated with regional brain volumes; and (v) clustering of the biomarker concentrations in ex-players would reflect traumatic encephalopathy syndrome (TES) classification in ex-players, the proposed clinical correlate of CTE.^16^

## Methods

The Advanced Brain Health Clinic study is a prospective cohort of retired elite professional contact sport athletes aligned to a clinic, self-referred due to brain health concerns.^17^ This includes age-matched controls, with no history of elite sporting participation, TBI or substantial RHI exposure. The methods are described elsewhere (and see online supplemental file).^2^ Informed consent was given by all participants and the Camberwell and St Giles research ethics committee approved the study (ref 7/Lo/2066). Exclusions for this sub-study major un-related neurological/psychiatric diagnosis. Participants underwent a detailed assessment including semistructured interview for career history, position, TBI exposure (BRAIN-Q^18^/Ohio State^19^), plasma sampling, physical assessment, 3T MRI and a TES consensus classification.^16^

Plasma was analysed using the NULISA CNS disease panel at Alamar Biosciences’ facility (Freemont, CA) according to standard operating procedures^10^ to produce normalised protein expression levels (NPX). Previously quantified biomarker data from the Quanterix Simoa (Single molecule array) iplatform were available in the cohort for comparison. T1 volumetric imaging data were acquired using a Siemens Skyra 3T scanner with FreeSurfer used to calculate regional brain volumes.^2^

Statistical analyses were performed in R (4.3.1; Rstudio 2023.09.0+463). Variables were inspected visually for normality. Group comparisons were performed using parametric methods, unless the normality assumption was violated. Missing data were addressed using a complete-cases methodology. Protein fold change was calculated from normalised protein expression values using the following calculation: 2^ˆ(NPXrugby−NPXcontrol)^. Differential expression analysis was performed with the Limma package using robust regression. FDR correction was performed throughout (p=0.05 threshold). Covariates of age, sex and BMI were included in regressions, and estimated total intracranial volume in brain imaging analyses. K-means clustering was performed using two clusters (see online supplemental file). NULISA-Simoa correlations were performed using Spearman’s, with the Simoa markers log_2_ transformed.

Data underlying this study are available from the corresponding author on reasonable request.

## Results

197 ex-players, aged 44 (median, IQR 39–51) years, of predominantly male sex (90%) and 33 controls were assessed. The median elite career duration was 10.0 (IQR 8–13) years, with 63% being former forwards (see table 1). Players were an average of 13.6 years (median, IQR 11.0) following retirement when assessed. NULISA proteomic data were available in all participants (online supplemental table 1).

**Table 1:** Demographics of ex-players and unexposed healthy controls

	*Rugby (all*)	*Rugby backs*	*Rugby forwards*	*Controls*	*P (rugby vs control*)*
	n=197	n=73	n=124	n=33	
Age (years)	44 (39, 51)	45 (39, 50)	44 (39, 52)	47 (40, 56)	0.14
Sex					0.071
Male	178 (90%)	64 (88%)	114 (92%)	26 (79%)	
Female	19 (9.6%)	9 (12%)	10 (8.1%)	7 (21%)	
BMI (kg/m^2^)†	29.0 (27.2, 32.6)	28.1 (26.4, 29.5)	30.1 (27.9, 34.8)	26.0 (23.2, 28.0)	<0.001
Player position					
Back	73 (37%)	73 (100%)	0 (0%)		
Forward	124 (63%)	0 (0%)	124 (100%)		
Elite career duration (years)†	10.0 (8.0, 13.0)	10.0 (7.0, 12.0)	11.0 (8.0, 13.0)		
Traumatic encephalopathy syndrome					
Present	24 (12%)	6 (8.2%)	18 (14.5%)		
Absent	173 (88%)	67 (91.8%)	106 (85.4%)		

* Wilcoxon rank sum test; Fisher’s exact test.

† Median (Q1, Q3); n (%)2

BMI, body mass index.

**Figure 1:**
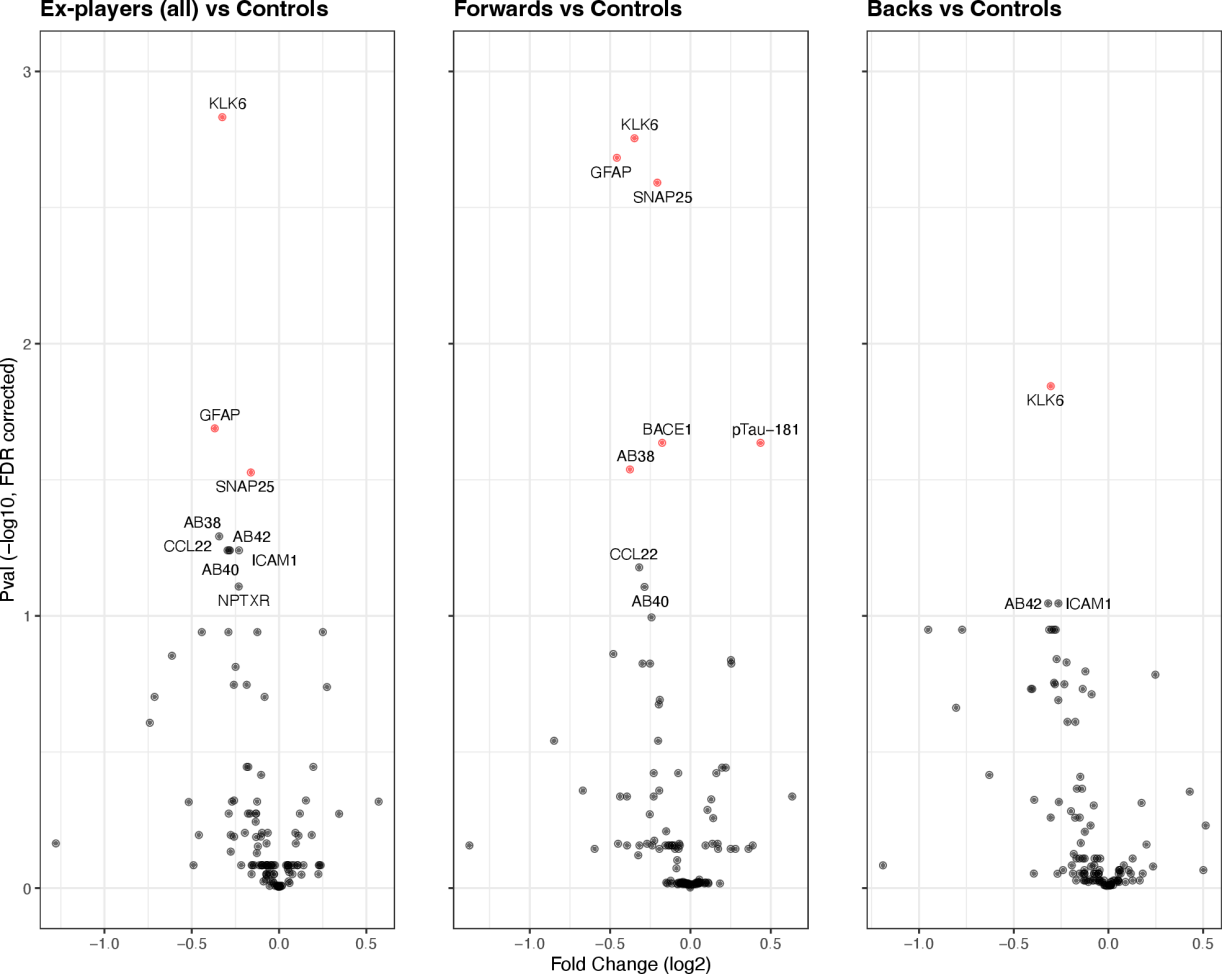
Plasma proteomic changes in ex-players and unexposed controls. Differential expression analysis across 124 proteins on NULISA platform in former elite rugby players and age/sex-matched unexposed controls. Red datapoints indicate significance at false discovery rate (FDR) corrected threshold of p=0.05. Aβ38, amyloid beta 38; GFAP, glial fibrillar acidic protein; KLK6, kallikrein-6; NULISA, NUcleic acid Linked Immuno-Sandwich Assa; pTau217, phospho-tau217; SNAP25, synaptosomal-associated protein 25.

Differential expression analysis demonstrated significant reductions in ex-players of plasma kallikrein-6 (KLK6, fold change (FC)=0.80 (95% CI 0.72 to 0.88), P_adj_=0.001), a pro-inflammatory protease; astroglial marker glial fibrillar acidic protein (GFAP, FC=0.77 (0.67–0.89), P_adj_=0.020); and synaptic transmission regulator synaptosomal-associated protein 25 (SNAP25, FC=0.89 0.84–0.95), P_adj_=0.030) vs controls (See figure 1 and online supplemental tables 1 and 2).

Assessing protein expression by player position, ex-forwards returned significantly raised phospho-tau181 (p-tau_181_, FC=1.35 (1.13–1.62), Padj=0.023) vs controls, alongside reductions in beta-site amyloid precursor protein cleaving enzyme 1 (BACE1, FC=0.89 (0.82–0.95)] P_adj_=0.023), amyloid beta-38 (Aβ38, FC=0.77 (0.66–0.90), P_adj_=0.029), KLK6 (FC=0.79 (0.71–0.87), P_adj_=0.002), GFAP (FC=0.73 (0.63–0.84), P_adj_=0.002) and SNAP25 (FC=0.87 (0.81–0.93), P_adj_=0.003). Only KLK6 was differentially expressed in backs vs controls (FC=0.81 (0.73–0.90), P_adj_=0.014), and there were no differences between forwards and backs (online supplemental tables 1 and 2). There were no significant proteomic correlates of career duration, nor of concussion load after correction for multiple comparisons.

Research criteria TES were met in 12 (24%) ex-players. Unbiased K-means clustering of ex-players using NULISA biomarker profiles did not produce a similar classification (K=2, 51.8%, adjusted Rand index=−0.001).

Regional brain volumes were quantified in 196 (99.5%) ex-players and all controls (online supplemental table 3). There were no significant associations between the concentrations of proteins identified in the discovery analysis and total grey, white matter, hippocampal or ventricular volume.

Correlations between plasma proteins quantified on both the NULISA and Simoa platforms were assessed, returning strong relationships for GFAP (*ρ=0.79)* and NfL *(ρ=0.83*) and moderate correlations for p-tau217 (*ρ=*0.56), amyloid beta 42 (*ρ=*0.57) and amyloid beta 40 (*ρ=*0.49) (all p<0.001).

## Discussion

Using a highly multiplexed central nervous system biomarker panel, we have identified distinctive plasma protein abnormalities in former elite rugby players. Ex-players had significant reductions in plasma KLK6, a serine protease with roles in amyloid precursor protein, tau, myelin basic protein and α-synuclein processing.^20^ Previous work has shown reductions of KLK6 in brain tissue of people with AD, and its expression patterns are notable for localisation to the cerebral vascular endothelium,^21^ of spatial importance in CTE.^3^

Ex-forwards showed more extensive proteomic change than ex-backs, raising the possibility of a dose-response relationship to RHI exposure. These changes included raised plasma p-tau181 and reductions in BACE1, amyloid beta-38, SNAP25 and GFAP. However, while player position did relate to protein expression, we did not observe a significant association with career duration or concussion load.

The identification on the NULISA platform of protein changes related to both amyloid (eg, BACE1, Aβ38) and tau (p-tau181) metabolism in ex-players extends our previous work in this cohort, which found elevations in plasma p-tau_217_ (Simoa platform). Our NULISA data suggest that ex-players’ p-tau_181_ levels were greater than p-tau_217_: the cause of this relative difference is uncertain, but it is not typical of AD profiles on this panel where the ratio is usually reversed.^22^ Furthermore, p-tau_217_ is typically deranged earlier in the AD process than p-tau_181_.^23^ One possibility is a peripheral source of phospho-tau, such as muscle or nerve, as has been hypothesised in amyotrophic lateral sclerosis, where substantial p-tau_181_ elevations are seen in plasma, but not CSF.^24 25^

Proteomic signatures on cluster analysis did not map well to research diagnostic TES classification. This may indicate that current criteria may not adequately capture underlying biological heterogeneity, or simply that our cohort is at an early/presymptomatic phase. Furthermore, we are uncertain of the basis of the observed reduction in plasma GFAP in ex-forwards, which contrasts with raised GFAP in acute^26^ TBI, chronic TBI^27^ and AD, where it is thought to reflect Aβ but not neurofibrillary tangle burden.^28^ Body habitus may influence concentrations,^29^ though this was accounted for in our analyses. In the event that this is not biological in origin, a further possibility is analytic variability related to use of the Alamar panel, although NULISA GFAP values were well correlated with previously performed Quanterix Simoa analyses (*ρ=0.79*). Likewise, reduced SNAP25 concentration is of uncertain significance here, contrasting with elevations in AD owing to synaptic degeneration.^30^

There are several limitations to this biomarker discovery work: research participants were predominantly male; hence, generalisability is uncertain and the control group was relatively small compared with the numbers of ex-players. This may have reduced sensitivity to small biological effects. This is a cross-sectional study, and longitudinal follow-up will help to clarify whether abnormalities evolve over time and relate to a clinical phenotype, benefiting from planned comparisons to canonical neurodegenerative diseases.

In conclusion, we have shown distinctive plasma biomarker changes in ex-players, implicating amyloid beta processing, with important implications for monitoring brain health in contact sport athletes, and highlighting the utility of large-scale proteomic panels. Longitudinal studies are essential to clarify the significance of these changes and to determine whether these midlife proteomic signatures predict clinical deterioration or represent valid clinical trial targets.

## Supplementary material

10.1136/jnnp-2025-336593 online supplemental file 1
