# Supplementary material for: Midlife plasma proteomic profiles indicate altered amyloid and tau processing in former elite rugby players
Source: J Neurol Neurosurg Psychiatry. 2025 Oct 5;97(5):e336593. doi: 10.1136/jnnp-2025-336593 (PMC13151487; doi:10.1136/jnnp-2025-336593)
Supplement: online supplemental file 1 [file jnnp-97-5-s001.docx]

**SUPPLEMENTARY MATERIALS**

**Midlife Plasma Proteomic Profiles Indicate Altered Amyloid and Tau Processing in Former Elite Rugby Players**

**SUPPLEMENTARY METHODS**

Study design: Briefly, all participants undergo a detailed clinical evaluation by a consultant neurologist, and additional research data are collected in consenting individuals, where written informed consent is given as per the Declaration of Helsinki. Players of either sex were eligible for inclusion. Inclusions for elite ex-rugby players were: retired England Men/Womens, Retired England 7s Men/Women, Retired Premiership players, Retired AP15s players or Retired Championship players. The latter three player types need to have been contracted for at least one full season (leagues represent the highest level of domestic men and women’s leagues, and the second highest men’s league in England). Elite career duration was defined as number of years at a premiership/international level.

Imaging: The following sequences were included: T1-weighted MPRAGE (1mm^3^ voxels, 160 slices, TR = 2300 ms, TE = 2.98 ms, GRAPPA = 2), T_2_ SWI (0.8 x 0.6 x 1.2mm voxels, 120 slices, TR = 28 ms, TE = 20 ms, GRAPPA = 2), T_2_ fluid-attenuated inversion recovery (FLAIR; 1mm^3^ voxels, 160 slices, TR = 5000 ms, TE = 395 ms, GRAPPA = 2), diffusion MRI (64 directions, 2mm^3^ voxel size, b = 1000 s/mm^3^, 4 b = 0 s/mm^2^, TR = 9500 ms, TE = 103ms).

Blood processing: Venous blood was sampled and centrifuged for 10 minutes at 2500g, prior to aliquoting and freezing at -80C. Plasma samples were randomised, plated and couriered to Alamar (Fremont, California) for quantification of CNS injury/neurodegenerative/inflammatory markers using the NULISA CNS disease 120 panel. Prior SIMOA data were available from the same study visit timepoint, comprising NfL, GFAP, Aß40 and Aß42 assessed on a Simoa-Neurology 4-plex-E panel, and p-tau_217_ using a Simoa ALZpath assay.

Cluster analysis: To investigate whether the high-dimensional biomarker profiles of ex-players naturally segregate into groups corresponding to clinically defined TES criteria, we performed an unsupervised clustering analysis using the 124 biomarkers. First, the biomarker data were extracted and standardized via z-score transformation to ensure that each feature contributed equally to the distance metrics used in clustering. We then applied k-means clustering with

k=2 , using 25 starts (reflecting the two clinical groups: "TES negative" and "TES positive") on the standardized data. To quantitatively assess the agreement between the unsupervised clusters and the clinically defined categories, we calculated the Adjusted Rand Index (ARI) using the flexclust R package.

**SUPPLEMENTARY TABLES**

**Supplementary Table 1. Protein concentrations and differential expression results**

|  | **Biomarker Concentrations** (NPX, Q1, Q3) | | | | **Differential Expression Results** | | | | | | | | | | | |
| --- | --- | --- | --- | --- | --- | --- | --- | --- | --- | --- | --- | --- | --- | --- | --- | --- |
|  | **Ex-players (all)** | **Forwards** | **Backs** | **Controls** | **Ex-players (all) vs healthy controls** | | | **Ex-forwards vs healthy controls** | | | **Ex-backs vs healthy controls** | | | **Ex-forwards vs backs** | | |
|  | N = 197 | N = 124 | N = 73 | N = 33 | Fold-change | P | Padj | Fold-change | P | Padj | Fold-change | P | Padj | Fold-change | P | Padj |
| **ACHE** | 14.09 (13.86, 14.29) | 14.08 (13.86, 14.27) | 14.13 (13.88, 14.30) | 14.04 (13.74, 14.21) | 1.04 (0.95-1.14) | 0.439 | 0.824 | 1.03 (0.93-1.14) | 0.521 | 0.949 | 1.04 (0.94-1.15) | 0.429 | 0.859 | 0.99 (0.92-1.07) | 0.848 | 0.973 |
| **AGRN** | 13.73 (13.53, 13.88) | 13.74 (13.56, 13.90) | 13.70 (13.50, 13.87) | 13.76 (13.67, 13.84) | 0.96 (0.89-1.03) | 0.217 | 0.627 | 0.95 (0.88-1.03) | 0.219 | 0.687 | 0.96 (0.89-1.04) | 0.283 | 0.779 | 0.99 (0.94-1.05) | 0.815 | 0.971 |
| **ANXA5** | 8.81 (8.04, 9.77) | 8.84 (8.27, 9.84) | 8.76 (7.67, 9.66) | 8.70 (8.00, 9.73) | 0.79 (0.68-0.91) | 0.002 | 0.051 | 0.77 (0.66-0.90) | 0.001 | 0.029 | 0.81 (0.69-0.94) | 0.007 | 0.112 | 0.96 (0.85-1.07) | 0.447 | 0.906 |
| **APOE** | 13.51 (13.25, 14.00) | 13.61 (13.32, 14.05) | 13.37 (13.14, 13.86) | 13.65 (13.18, 14.09) | 0.82 (0.72-0.93) | 0.003 | 0.057 | 0.82 (0.71-0.94) | 0.005 | 0.078 | 0.82 (0.72-0.94) | 0.006 | 0.112 | 0.99 (0.90-1.10) | 0.917 | 0.992 |
| **APOE4** | 0.9 (0.0, 12.8) | 0.8 (0.0, 12.9) | 1.0 (0.0, 12.7) | 1.4 (0.0, 12.8) | 0.82 (0.72-0.93) | 0.003 | 0.057 | 0.84 (0.73-0.97) | 0.016 | 0.150 | 0.80 (0.70-0.92) | 0.002 | 0.090 | 1.05 (0.95-1.16) | 0.378 | 0.837 |
| **ARSA** | 9.64 (9.01, 10.45) | 9.64 (9.17, 10.48) | 9.58 (8.97, 10.38) | 9.58 (8.84, 10.39) | 0.83 (0.56-1.21) | 0.326 | 0.735 | 0.80 (0.53-1.22) | 0.298 | 0.697 | 0.85 (0.56-1.28) | 0.425 | 0.859 | 0.95 (0.70-1.28) | 0.724 | 0.930 |
| **AB38** | 10.90 (10.61, 11.24) | 10.89 (10.61, 11.27) | 10.93 (10.60, 11.18) | 11.31 (10.91, 11.56) | 0.88 (0.77-1.01) | 0.069 | 0.359 | 0.90 (0.77-1.05) | 0.17 | 0.619 | 0.86 (0.74-1.00) | 0.047 | 0.245 | 1.05 (0.94-1.17) | 0.415 | 0.902 |
| **AB40** | 13.79 (13.46, 14.09) | 13.79 (13.49, 14.11) | 13.79 (13.43, 14.01) | 14.00 (13.87, 14.15) | 0.41 (0.08-2.16) | 0.293 | 0.685 | 0.39 (0.06-2.32) | 0.296 | 0.697 | 0.44 (0.07-2.57) | 0.36 | 0.825 | 0.88 (0.24-3.21) | 0.845 | 0.973 |
| **AB42** | 14.25 (13.98, 14.45) | 14.25 (14.02, 14.45) | 14.23 (13.90, 14.45) | 14.54 (14.31, 14.73) | 0.98 (0.75-1.30) | 0.911 | 0.981 | 0.98 (0.73-1.33) | 0.915 | 0.969 | 0.98 (0.73-1.32) | 0.919 | 0.976 | 1.00 (0.81-1.24) | 0.994 | 0.995 |
| **BACE1** | 13.54 (13.37, 13.71) | 13.49 (13.34, 13.64) | 13.68 (13.46, 13.81) | 13.72 (13.62, 13.83) | 0.92 (0.86-0.98) | 0.011 | 0.115 | 0.89 (0.82-0.95) | <0.001 | 0.023 | 0.95 (0.88-1.02) | 0.132 | 0.497 | 0.93 (0.89-0.98) | 0.009 | 0.405 |
| **BASP1** | 6.81 (6.12, 7.90) | 6.72 (6.11, 7.90) | 6.94 (6.14, 8.14) | 6.92 (6.26, 8.38) | 0.94 (0.56-1.59) | 0.816 | 0.945 | 0.97 (0.55-1.71) | 0.914 | 0.969 | 0.91 (0.52-1.60) | 0.751 | 0.938 | 1.06 (0.70-1.60) | 0.776 | 0.953 |
| **BDNF** | 12.44 (11.44, 13.55) | 12.53 (11.48, 14.02) | 12.26 (11.32, 13.29) | 12.96 (11.77, 14.22) | 0.61 (0.39-0.96) | 0.032 | 0.198 | 0.73 (0.45-1.19) | 0.206 | 0.687 | 0.52 (0.32-0.83) | 0.007 | 0.112 | 1.41 (1.00-2.01) | 0.052 | 0.576 |
| **CALB2** | 11.30 (11.04, 11.64) | 11.37 (11.11, 11.67) | 11.19 (10.96, 11.56) | 11.29 (11.00, 11.71) | 0.91 (0.80-1.04) | 0.156 | 0.533 | 0.94 (0.82-1.07) | 0.342 | 0.718 | 0.89 (0.78-1.02) | 0.096 | 0.432 | 1.05 (0.95-1.16) | 0.333 | 0.826 |
| **CCL11** | 13.74 (13.49, 14.04) | 13.75 (13.50, 14.08) | 13.70 (13.45, 13.99) | 13.88 (13.61, 14.12) | 0.92 (0.82-1.02) | 0.116 | 0.481 | 0.93 (0.83-1.05) | 0.247 | 0.697 | 0.90 (0.80-1.01) | 0.082 | 0.390 | 1.03 (0.95-1.13) | 0.435 | 0.906 |
| **CCL13** | 13.04 (12.71, 13.44) | 13.14 (12.76, 13.58) | 12.93 (12.66, 13.31) | 13.08 (12.74, 13.40) | 0.95 (0.82-1.11) | 0.526 | 0.824 | 1.01 (0.85-1.19) | 0.935 | 0.969 | 0.90 (0.77-1.07) | 0.226 | 0.683 | 1.11 (0.99-1.26) | 0.078 | 0.654 |
| **CCL17** | 12.48 (11.82, 13.19) | 12.53 (11.82, 13.53) | 12.42 (11.86, 12.78) | 12.71 (11.90, 13.48) | 0.83 (0.60-1.13) | 0.234 | 0.638 | 0.90 (0.64-1.27) | 0.549 | 0.955 | 0.76 (0.54-1.07) | 0.115 | 0.474 | 1.18 (0.92-1.51) | 0.184 | 0.749 |
| **CCL2** | 13.04 (12.75, 13.32) | 13.07 (12.78, 13.43) | 13.00 (12.68, 13.23) | 13.02 (12.76, 13.42) | 0.91 (0.80-1.04) | 0.179 | 0.571 | 0.92 (0.80-1.07) | 0.287 | 0.697 | 0.90 (0.78-1.04) | 0.156 | 0.551 | 1.03 (0.92-1.14) | 0.642 | 0.912 |
| **CCL22** | 13.10 (12.80, 13.36) | 13.12 (12.82, 13.37) | 13.01 (12.78, 13.32) | 13.19 (13.05, 13.56) | 0.81 (0.71-0.94) | 0.004 | 0.057 | 0.80 (0.69-0.93) | 0.004 | 0.066 | 0.83 (0.71-0.96) | 0.012 | 0.144 | 0.97 (0.87-1.08) | 0.564 | 0.912 |
| **CCL26** | 12.31 (12.04, 12.65) | 12.29 (12.04, 12.67) | 12.34 (12.04, 12.62) | 12.31 (12.02, 12.71) | 0.99 (0.84-1.17) | 0.931 | 0.981 | 0.98 (0.82-1.17) | 0.821 | 0.962 | 1.00 (0.84-1.20) | 0.958 | 0.977 | 0.97 (0.86-1.11) | 0.7 | 0.924 |
| **CCL3** | 12.33 (12.03, 12.70) | 12.34 (12.12, 12.80) | 12.21 (11.97, 12.53) | 12.22 (11.97, 12.70) | 0.95 (0.80-1.13) | 0.563 | 0.824 | 0.95 (0.79-1.15) | 0.596 | 0.955 | 0.95 (0.79-1.14) | 0.586 | 0.886 | 1.00 (0.87-1.15) | 0.992 | 0.995 |
| **CCL4** | 12.54 (12.04, 13.01) | 12.67 (11.97, 13.02) | 12.41 (12.06, 12.97) | 12.37 (11.89, 12.99) | 0.95 (0.73-1.24) | 0.707 | 0.889 | 0.94 (0.71-1.26) | 0.69 | 0.962 | 0.96 (0.72-1.27) | 0.761 | 0.938 | 0.99 (0.80-1.21) | 0.891 | 0.986 |
| **CD40LG** | 11.03 (10.62, 11.35) | 11.09 (10.74, 11.42) | 10.87 (10.55, 11.29) | 10.96 (10.66, 11.45) | 0.93 (0.78-1.12) | 0.458 | 0.824 | 0.97 (0.80-1.18) | 0.793 | 0.962 | 0.90 (0.74-1.09) | 0.277 | 0.779 | 1.08 (0.94-1.25) | 0.262 | 0.772 |
| **CD63** | 12.74 (12.40, 13.07) | 12.73 (12.41, 13.09) | 12.74 (12.36, 13.06) | 12.35 (12.25, 12.82) | 1.15 (0.99-1.33) | 0.067 | 0.359 | 1.16 (0.99-1.36) | 0.058 | 0.361 | 1.13 (0.97-1.32) | 0.126 | 0.487 | 1.03 (0.92-1.16) | 0.594 | 0.912 |
| **CHI3L1** | 12.37 (12.04, 12.89) | 12.37 (12.01, 12.91) | 12.41 (12.04, 12.88) | 12.49 (12.13, 12.97) | 0.87 (0.70-1.08) | 0.216 | 0.627 | 0.86 (0.68-1.08) | 0.189 | 0.670 | 0.89 (0.71-1.12) | 0.318 | 0.779 | 0.96 (0.81-1.14) | 0.649 | 0.912 |
| **CHIT1** | 13.44 (12.88, 13.96) | 13.48 (12.91, 13.98) | 13.32 (12.67, 13.77) | 13.52 (12.99, 14.10) | 0.71 (0.32-1.58) | 0.403 | 0.824 | 0.66 (0.28-1.57) | 0.347 | 0.718 | 0.76 (0.32-1.79) | 0.53 | 0.885 | 0.87 (0.47-1.62) | 0.658 | 0.912 |
| **CNTN2** | 14.01 (13.47, 14.62) | 13.99 (13.42, 14.57) | 14.03 (13.55, 14.64) | 14.40 (13.85, 14.87) | 0.91 (0.70-1.17) | 0.442 | 0.824 | 0.87 (0.66-1.15) | 0.338 | 0.718 | 0.93 (0.71-1.23) | 0.622 | 0.930 | 0.94 (0.77-1.14) | 0.513 | 0.912 |
| **CRH** | 14.65 (13.80, 15.34) | 14.54 (13.65, 15.23) | 14.82 (14.27, 15.53) | 14.86 (14.06, 15.84) | 0.90 (0.69-1.17) | 0.422 | 0.824 | 0.85 (0.64-1.13) | 0.259 | 0.697 | 0.94 (0.71-1.25) | 0.689 | 0.938 | 0.90 (0.73-1.11) | 0.31 | 0.801 |
| **CRP** | 11.52 (10.86, 11.93) | 11.54 (11.09, 11.98) | 11.27 (10.68, 11.84) | 11.50 (10.93, 11.99) | 0.84 (0.67-1.04) | 0.103 | 0.477 | 0.84 (0.67-1.06) | 0.138 | 0.536 | 0.83 (0.66-1.05) | 0.121 | 0.483 | 1.01 (0.85-1.19) | 0.944 | 0.992 |
| **CSF2** | 12.89 (12.64, 13.18) | 12.95 (12.64, 13.22) | 12.86 (12.65, 13.09) | 12.92 (12.50, 13.16) | 1.04 (0.88-1.24) | 0.648 | 0.873 | 1.05 (0.87-1.26) | 0.635 | 0.955 | 1.04 (0.86-1.25) | 0.704 | 0.938 | 1.01 (0.88-1.16) | 0.89 | 0.986 |
| **CST3** | 13.77 (13.67, 13.87) | 13.78 (13.69, 13.88) | 13.76 (13.64, 13.87) | 13.79 (13.67, 13.92) | 0.94 (0.90-0.99) | 0.032 | 0.198 | 0.95 (0.90-1.00) | 0.067 | 0.378 | 0.94 (0.89-0.99) | 0.031 | 0.194 | 1.01 (0.97-1.05) | 0.676 | 0.912 |
| **CX3CL1** | 13.83 (13.64, 14.03) | 13.87 (13.66, 14.03) | 13.79 (13.61, 14.02) | 13.80 (13.63, 14.02) | 0.98 (0.91-1.06) | 0.592 | 0.824 | 0.99 (0.91-1.08) | 0.825 | 0.962 | 0.97 (0.89-1.05) | 0.45 | 0.859 | 1.02 (0.96-1.09) | 0.468 | 0.906 |
| **CXCL1** | 12.41 (11.89, 12.94) | 12.37 (11.87, 13.01) | 12.47 (11.94, 12.90) | 12.40 (11.96, 13.32) | 0.82 (0.62-1.08) | 0.155 | 0.533 | 0.83 (0.61-1.12) | 0.219 | 0.687 | 0.81 (0.60-1.09) | 0.16 | 0.551 | 1.02 (0.83-1.27) | 0.826 | 0.973 |
| **CXCL10** | 12.43 (12.01, 12.90) | 12.54 (12.13, 13.02) | 12.17 (11.89, 12.66) | 12.47 (12.13, 12.69) | 0.93 (0.76-1.15) | 0.508 | 0.824 | 0.99 (0.79-1.24) | 0.948 | 0.969 | 0.88 (0.71-1.10) | 0.26 | 0.749 | 1.13 (0.96-1.32) | 0.147 | 0.749 |
| **CXCL8** | 11.82 (11.57, 12.10) | 11.83 (11.63, 12.10) | 11.81 (11.52, 12.07) | 11.79 (11.63, 12.26) | 0.91 (0.78-1.07) | 0.262 | 0.649 | 0.91 (0.76-1.08) | 0.275 | 0.697 | 0.92 (0.77-1.09) | 0.318 | 0.779 | 0.99 (0.87-1.12) | 0.883 | 0.986 |
| **ENO2** | 11.25 (10.93, 11.57) | 11.22 (10.90, 11.55) | 11.27 (10.98, 11.64) | 11.10 (10.92, 11.65) | 0.95 (0.83-1.10) | 0.503 | 0.824 | 0.91 (0.78-1.07) | 0.249 | 0.697 | 0.99 (0.85-1.15) | 0.889 | 0.975 | 0.92 (0.83-1.03) | 0.16 | 0.749 |
| **FABP3** | 13.68 (13.39, 14.08) | 13.75 (13.45, 14.22) | 13.61 (13.28, 13.83) | 13.53 (13.19, 13.80) | 1.08 (0.95-1.23) | 0.243 | 0.642 | 1.15 (1.00-1.32) | 0.055 | 0.361 | 1.02 (0.89-1.18) | 0.734 | 0.938 | 1.12 (1.01-1.24) | 0.029 | 0.454 |
| **FCN2** | 14.10 (13.77, 14.45) | 14.12 (13.82, 14.45) | 14.10 (13.72, 14.44) | 14.08 (13.53, 14.33) | 0.99 (0.86-1.15) | 0.914 | 0.981 | 0.98 (0.84-1.15) | 0.794 | 0.962 | 1.00 (0.86-1.17) | 0.962 | 0.977 | 0.98 (0.87-1.09) | 0.67 | 0.912 |
| **FGF2** | 11.46 (10.66, 12.45) | 11.56 (10.83, 12.48) | 11.40 (10.60, 12.22) | 11.46 (10.80, 11.89) | 0.95 (0.71-1.27) | 0.739 | 0.898 | 1.01 (0.74-1.39) | 0.936 | 0.969 | 0.90 (0.66-1.23) | 0.503 | 0.885 | 1.13 (0.90-1.41) | 0.305 | 0.801 |
| **FLT1** | 15.06 (14.81, 15.30) | 15.08 (14.82, 15.32) | 15.03 (14.78, 15.28) | 15.10 (14.79, 15.31) | 0.98 (0.88-1.10) | 0.717 | 0.889 | 1.00 (0.88-1.12) | 0.943 | 0.969 | 0.97 (0.86-1.09) | 0.56 | 0.886 | 1.03 (0.94-1.13) | 0.485 | 0.912 |
| **FOLR1** | 12.64 (12.38, 12.85) | 12.65 (12.38, 12.87) | 12.63 (12.38, 12.82) | 12.77 (12.56, 13.00) | 0.97 (0.88-1.06) | 0.501 | 0.824 | 0.99 (0.90-1.09) | 0.838 | 0.962 | 0.95 (0.86-1.05) | 0.311 | 0.779 | 1.04 (0.97-1.12) | 0.27 | 0.779 |
| **GDF15** | 13.79 (13.22, 14.24) | 13.79 (13.17, 14.26) | 13.79 (13.35, 14.19) | 14.21 (13.69, 14.47) | 0.73 (0.43-1.23) | 0.234 | 0.638 | 0.56 (0.32-0.97) | 0.04 | 0.288 | 0.93 (0.53-1.62) | 0.794 | 0.949 | 0.60 (0.40-0.90) | 0.013 | 0.405 |
| **GDI1** | 12.66 (12.51, 12.75) | 12.67 (12.53, 12.77) | 12.61 (12.49, 12.72) | 12.61 (12.54, 12.76) | 0.98 (0.93-1.04) | 0.556 | 0.824 | 1.00 (0.94-1.06) | 0.974 | 0.982 | 0.97 (0.92-1.03) | 0.306 | 0.779 | 1.03 (0.99-1.07) | 0.176 | 0.749 |
| **GDNF** | 10.78 (9.81, 11.83) | 10.87 (9.94, 11.86) | 10.55 (9.73, 11.79) | 10.52 (9.70, 11.95) | 1.17 (0.74-1.85) | 0.489 | 0.824 | 1.31 (0.80-2.14) | 0.284 | 0.697 | 1.06 (0.65-1.73) | 0.8 | 0.949 | 1.23 (0.86-1.75) | 0.256 | 0.772 |
| **GFAP** | 13.89 (13.57, 14.21) | 13.82 (13.55, 14.13) | 14.00 (13.61, 14.38) | 14.22 (13.96, 14.53) | 0.77 (0.67-0.89) | <0.001 | 0.020 | 0.73 (0.63-0.84) | <0.001 | 0.002 | 0.82 (0.71-0.95) | 0.008 | 0.112 | 0.89 (0.80-0.99) | 0.029 | 0.454 |
| **GOT1** | 11.96 (11.67, 12.26) | 11.96 (11.66, 12.28) | 11.96 (11.69, 12.25) | 11.88 (11.44, 12.22) | 1.04 (0.90-1.19) | 0.618 | 0.852 | 1.04 (0.89-1.21) | 0.631 | 0.955 | 1.03 (0.89-1.20) | 0.654 | 0.938 | 1.00 (0.90-1.12) | 0.958 | 0.995 |
| **HBA1** | 7.77 (6.93, 9.42) | 7.82 (7.12, 9.45) | 7.59 (6.67, 9.38) | 7.01 (6.26, 8.62) | 1.48 (0.91-2.43) | 0.114 | 0.481 | 1.55 (0.91-2.64) | 0.106 | 0.461 | 1.43 (0.84-2.41) | 0.183 | 0.589 | 1.09 (0.74-1.59) | 0.672 | 0.912 |
| **HTT** | 12.54 (11.92, 13.18) | 12.59 (11.96, 13.27) | 12.39 (11.82, 13.16) | 12.67 (11.67, 12.90) | 0.97 (0.74-1.26) | 0.809 | 0.945 | 0.97 (0.72-1.29) | 0.822 | 0.962 | 0.97 (0.73-1.29) | 0.823 | 0.949 | 1.00 (0.81-1.23) | 0.995 | 0.995 |
| **ICAM1** | 13.30 (13.09, 13.50) | 13.34 (13.12, 13.56) | 13.24 (13.05, 13.39) | 13.39 (13.20, 13.61) | 0.85 (0.77-0.95) | 0.004 | 0.057 | 0.88 (0.78-0.98) | 0.025 | 0.203 | 0.83 (0.74-0.93) | 0.002 | 0.090 | 1.05 (0.97-1.14) | 0.228 | 0.757 |
| **IFNG** | 11.02 (10.64, 11.72) | 11.04 (10.67, 11.73) | 10.99 (10.55, 11.71) | 11.02 (10.59, 11.46) | 1.10 (0.78-1.56) | 0.58 | 0.824 | 1.02 (0.71-1.49) | 0.9 | 0.969 | 1.18 (0.81-1.70) | 0.383 | 0.833 | 0.87 (0.66-1.14) | 0.308 | 0.801 |
| **IGF1R** | 13.74 (13.62, 13.84) | 13.74 (13.63, 13.83) | 13.74 (13.61, 13.84) | 13.76 (13.62, 13.84) | 1.00 (0.96-1.05) | 0.921 | 0.981 | 1.00 (0.95-1.05) | 0.951 | 0.969 | 1.00 (0.96-1.05) | 0.904 | 0.975 | 1.00 (0.96-1.03) | 0.937 | 0.992 |
| **IGFBP7** | 13.95 (13.77, 14.07) | 13.97 (13.81, 14.09) | 13.91 (13.72, 14.02) | 13.86 (13.79, 14.05) | 1.00 (0.93-1.08) | 0.939 | 0.981 | 1.02 (0.94-1.11) | 0.566 | 0.955 | 0.98 (0.91-1.07) | 0.696 | 0.938 | 1.04 (0.98-1.10) | 0.185 | 0.749 |
| **IL10** | 12.04 (11.65, 12.49) | 12.10 (11.72, 12.54) | 12.01 (11.59, 12.36) | 11.94 (11.70, 12.48) | 0.98 (0.80-1.19) | 0.807 | 0.945 | 0.96 (0.77-1.19) | 0.693 | 0.962 | 0.99 (0.80-1.23) | 0.941 | 0.977 | 0.96 (0.82-1.13) | 0.655 | 0.912 |
| **IL12p70** | 13.58 (13.12, 14.01) | 13.66 (13.10, 14.01) | 13.53 (13.15, 14.02) | 13.72 (13.15, 13.98) | 0.98 (0.80-1.20) | 0.843 | 0.959 | 0.99 (0.79-1.24) | 0.953 | 0.969 | 0.97 (0.78-1.21) | 0.764 | 0.938 | 1.03 (0.87-1.21) | 0.742 | 0.930 |
| **IL13** | 13.85 (13.18, 14.44) | 13.85 (13.09, 14.43) | 13.92 (13.22, 14.47) | 13.91 (13.47, 14.48) | 0.84 (0.61-1.14) | 0.256 | 0.648 | 0.76 (0.55-1.06) | 0.106 | 0.461 | 0.91 (0.66-1.27) | 0.582 | 0.886 | 0.83 (0.66-1.06) | 0.137 | 0.749 |
| **IL15** | 13.52 (13.34, 13.74) | 13.55 (13.34, 13.69) | 13.47 (13.33, 13.82) | 13.48 (13.35, 13.64) | 1.03 (0.94-1.13) | 0.47 | 0.824 | 1.03 (0.93-1.14) | 0.578 | 0.955 | 1.04 (0.94-1.15) | 0.437 | 0.859 | 0.99 (0.92-1.06) | 0.77 | 0.953 |
| **IL16** | 13.50 (13.23, 13.72) | 13.57 (13.30, 13.77) | 13.37 (13.15, 13.61) | 13.47 (13.31, 13.62) | 0.93 (0.84-1.04) | 0.214 | 0.627 | 0.96 (0.86-1.08) | 0.536 | 0.955 | 0.91 (0.81-1.02) | 0.097 | 0.432 | 1.06 (0.98-1.15) | 0.158 | 0.749 |
| **IL17A** | 12.21 (11.78, 12.94) | 12.21 (11.78, 12.88) | 12.13 (11.77, 12.97) | 12.51 (12.06, 13.06) | 0.86 (0.54-1.37) | 0.525 | 0.824 | 0.80 (0.48-1.32) | 0.379 | 0.758 | 0.92 (0.56-1.51) | 0.744 | 0.938 | 0.87 (0.60-1.25) | 0.44 | 0.906 |
| **IL18** | 11.42 (11.05, 11.73) | 11.44 (11.06, 11.81) | 11.37 (10.96, 11.67) | 11.38 (11.05, 11.60) | 0.96 (0.83-1.12) | 0.625 | 0.852 | 0.98 (0.83-1.16) | 0.798 | 0.962 | 0.95 (0.80-1.12) | 0.525 | 0.885 | 1.03 (0.92-1.16) | 0.607 | 0.912 |
| **IL1B** | 10.77 (10.68, 10.86) | 10.78 (10.68, 10.86) | 10.74 (10.68, 10.83) | 10.75 (10.71, 10.89) | 0.99 (0.92-1.08) | 0.874 | 0.981 | 0.98 (0.90-1.07) | 0.592 | 0.955 | 1.01 (0.93-1.10) | 0.834 | 0.949 | 0.97 (0.91-1.03) | 0.304 | 0.801 |
| **IL2** | 13.19 (12.88, 13.56) | 13.20 (12.88, 13.56) | 13.19 (12.89, 13.52) | 13.36 (13.03, 13.67) | 0.92 (0.76-1.10) | 0.336 | 0.744 | 0.85 (0.70-1.04) | 0.107 | 0.461 | 0.98 (0.81-1.18) | 0.804 | 0.949 | 0.87 (0.76-1.00) | 0.059 | 0.576 |
| **IL33** | 12.89 (12.64, 13.24) | 12.95 (12.72, 13.31) | 12.82 (12.55, 13.04) | 12.92 (12.73, 13.22) | 0.88 (0.78-1.01) | 0.066 | 0.359 | 0.92 (0.80-1.06) | 0.268 | 0.697 | 0.85 (0.74-0.98) | 0.023 | 0.178 | 1.09 (0.98-1.20) | 0.113 | 0.736 |
| **IL4** | 12.34 (12.02, 12.64) | 12.36 (12.00, 12.73) | 12.29 (12.05, 12.51) | 12.57 (12.09, 12.85) | 0.89 (0.76-1.05) | 0.157 | 0.533 | 0.96 (0.81-1.14) | 0.639 | 0.955 | 0.83 (0.70-0.99) | 0.035 | 0.204 | 1.15 (1.02-1.31) | 0.025 | 0.454 |
| **IL5** | 13.24 (12.63, 14.07) | 13.21 (12.54, 14.04) | 13.33 (12.70, 14.14) | 13.10 (12.54, 13.44) | 1.27 (0.91-1.78) | 0.164 | 0.534 | 1.19 (0.83-1.71) | 0.348 | 0.718 | 1.35 (0.94-1.93) | 0.103 | 0.442 | 0.88 (0.68-1.15) | 0.352 | 0.837 |
| **IL6** | 11.66 (11.16, 12.29) | 11.74 (11.19, 12.40) | 11.49 (11.07, 12.14) | 11.85 (11.29, 12.44) | 0.74 (0.58-0.93) | 0.011 | 0.115 | 0.72 (0.55-0.93) | 0.011 | 0.138 | 0.75 (0.58-0.97) | 0.028 | 0.186 | 0.95 (0.79-1.15) | 0.598 | 0.912 |
| **IL6R** | 13.86 (13.55, 14.11) | 13.91 (13.65, 14.12) | 13.77 (13.53, 14.04) | 13.73 (13.62, 14.20) | 0.93 (0.82-1.05) | 0.251 | 0.647 | 0.94 (0.83-1.08) | 0.401 | 0.790 | 0.92 (0.80-1.05) | 0.2 | 0.621 | 1.03 (0.93-1.13) | 0.555 | 0.912 |
| **IL7** | 13.26 (12.82, 13.84) | 13.45 (12.95, 14.13) | 13.16 (12.72, 13.54) | 13.44 (12.85, 14.28) | 0.83 (0.66-1.05) | 0.115 | 0.481 | 0.92 (0.72-1.18) | 0.5 | 0.940 | 0.76 (0.59-0.97) | 0.027 | 0.186 | 1.21 (1.01-1.45) | 0.036 | 0.491 |
| **IL9** | 13.22 (12.78, 13.79) | 13.18 (12.82, 13.77) | 13.22 (12.69, 13.80) | 13.21 (12.81, 13.61) | 1.14 (0.92-1.41) | 0.237 | 0.638 | 1.13 (0.89-1.42) | 0.316 | 0.718 | 1.15 (0.91-1.45) | 0.234 | 0.692 | 0.98 (0.83-1.16) | 0.812 | 0.971 |
| **KDR** | 14.02 (13.26, 14.34) | 14.01 (13.25, 14.37) | 14.03 (13.39, 14.29) | 13.73 (13.23, 14.31) | 1.04 (0.70-1.56) | 0.839 | 0.959 | 1.08 (0.70-1.67) | 0.726 | 0.962 | 1.01 (0.66-1.55) | 0.969 | 0.977 | 1.07 (0.78-1.47) | 0.665 | 0.912 |
| **KLK6** | 13.21 (12.97, 13.45) | 13.21 (12.93, 13.43) | 13.27 (13.04, 13.48) | 13.51 (13.34, 13.83) | 0.80 (0.72-0.88) | <0.001 | 0.001 | 0.79 (0.71-0.87) | <0.001 | 0.002 | 0.81 (0.73-0.90) | <0.001 | 0.014 | 0.97 (0.90-1.05) | 0.436 | 0.906 |
| **MAPT** | 13.17 (12.89, 13.48) | 13.22 (12.94, 13.54) | 13.04 (12.85, 13.31) | 12.94 (12.76, 13.36) | 1.00 (0.86-1.17) | 0.961 | 0.988 | 1.04 (0.89-1.22) | 0.619 | 0.955 | 0.97 (0.83-1.14) | 0.711 | 0.938 | 1.07 (0.96-1.21) | 0.232 | 0.757 |
| **MDH1** | 11.57 (11.11, 12.34) | 11.57 (11.11, 12.42) | 11.58 (11.08, 12.19) | 11.27 (10.63, 11.98) | 1.08 (0.84-1.38) | 0.565 | 0.824 | 1.06 (0.81-1.39) | 0.647 | 0.955 | 1.09 (0.83-1.42) | 0.545 | 0.885 | 0.98 (0.81-1.19) | 0.847 | 0.973 |
| **MME** | 10.06 (8.92, 11.25) | 10.52 (9.00, 11.77) | 9.50 (8.51, 10.52) | 9.83 (8.92, 11.06) | 0.70 (0.44-1.10) | 0.121 | 0.483 | 0.76 (0.47-1.24) | 0.273 | 0.697 | 0.65 (0.40-1.05) | 0.077 | 0.384 | 1.18 (0.83-1.68) | 0.368 | 0.837 |
| **MSLN** | 13.52 (12.93, 14.05) | 13.63 (13.00, 14.15) | 13.32 (12.90, 13.80) | 13.58 (12.68, 14.19) | 1.00 (0.81-1.25) | 0.981 | 0.988 | 1.07 (0.84-1.35) | 0.584 | 0.955 | 0.95 (0.75-1.20) | 0.645 | 0.938 | 1.13 (0.95-1.34) | 0.166 | 0.749 |
| **NEFH** | 17.59 (15.69, 20.31) | 17.41 (15.63, 19.87) | 18.10 (15.93, 20.74) | 17.20 (15.54, 20.14) | 1.17 (0.53-2.56) | 0.696 | 0.889 | 0.95 (0.41-2.21) | 0.899 | 0.969 | 1.42 (0.61-3.27) | 0.414 | 0.859 | 0.67 (0.36-1.23) | 0.196 | 0.749 |
| **NEFL** | 13.68 (13.41, 14.04) | 13.65 (13.41, 14.04) | 13.72 (13.41, 14.06) | 13.80 (13.52, 14.11) | 1.01 (0.87-1.18) | 0.887 | 0.981 | 1.02 (0.86-1.20) | 0.858 | 0.969 | 1.01 (0.85-1.19) | 0.928 | 0.976 | 1.01 (0.89-1.14) | 0.901 | 0.989 |
| **NGF** | 9.25 (9.03, 9.49) | 9.27 (9.00, 9.53) | 9.21 (9.06, 9.37) | 9.07 (8.81, 9.37) | 1.19 (1.04-1.36) | 0.01 | 0.115 | 1.19 (1.03-1.38) | 0.016 | 0.150 | 1.19 (1.03-1.37) | 0.017 | 0.164 | 1.00 (0.91-1.11) | 0.932 | 0.992 |
| **NPTX1** | 13.41 (13.13, 13.67) | 13.43 (13.15, 13.73) | 13.35 (13.07, 13.65) | 13.51 (13.22, 13.80) | 0.96 (0.84-1.09) | 0.508 | 0.824 | 0.98 (0.84-1.13) | 0.738 | 0.962 | 0.94 (0.81-1.08) | 0.383 | 0.833 | 1.04 (0.94-1.15) | 0.465 | 0.906 |
| **NPTX2** | 14.30 (14.01, 14.55) | 14.29 (13.96, 14.54) | 14.35 (14.06, 14.59) | 14.29 (14.09, 14.68) | 0.98 (0.89-1.08) | 0.698 | 0.889 | 0.95 (0.85-1.06) | 0.353 | 0.718 | 1.01 (0.91-1.12) | 0.874 | 0.975 | 0.94 (0.87-1.02) | 0.134 | 0.749 |
| **NPTXR** | 12.01 (11.65, 12.25) | 11.93 (11.60, 12.20) | 12.10 (11.83, 12.28) | 12.30 (11.99, 12.54) | 0.85 (0.76-0.95) | 0.006 | 0.078 | 0.85 (0.75-0.96) | 0.007 | 0.101 | 0.86 (0.76-0.97) | 0.013 | 0.148 | 0.99 (0.90-1.08) | 0.743 | 0.930 |
| **NPY** | 15.38 (15.08, 15.67) | 15.34 (15.03, 15.65) | 15.39 (15.16, 15.71) | 15.53 (15.37, 15.74) | 0.91 (0.81-1.03) | 0.153 | 0.533 | 0.87 (0.76-0.99) | 0.042 | 0.288 | 0.95 (0.84-1.09) | 0.488 | 0.885 | 0.91 (0.83-1.00) | 0.06 | 0.576 |
| **NRGN** | 12.56 (11.14, 13.49) | 12.63 (11.31, 13.56) | 12.18 (10.93, 13.23) | 11.81 (10.60, 13.45) | 1.18 (0.74-1.87) | 0.48 | 0.824 | 1.28 (0.78-2.11) | 0.323 | 0.718 | 1.09 (0.67-1.79) | 0.723 | 0.938 | 1.18 (0.82-1.68) | 0.376 | 0.837 |
| **Oligo.SNCA** | 12.12 (11.34, 13.12) | 12.16 (11.37, 13.12) | 12.00 (11.23, 13.04) | 11.66 (11.25, 12.87) | 1.09 (0.66-1.80) | 0.727 | 0.893 | 1.06 (0.62-1.83) | 0.826 | 0.962 | 1.12 (0.66-1.91) | 0.673 | 0.938 | 0.95 (0.64-1.40) | 0.785 | 0.954 |
| **PARK7** | 12.33 (11.99, 12.83) | 12.32 (11.93, 12.83) | 12.36 (12.01, 12.83) | 12.39 (11.97, 12.67) | 1.00 (0.81-1.23) | 0.977 | 0.988 | 0.97 (0.77-1.23) | 0.826 | 0.962 | 1.02 (0.81-1.28) | 0.88 | 0.975 | 0.96 (0.81-1.13) | 0.61 | 0.912 |
| **PDGFRB** | 13.94 (13.66, 14.29) | 14.01 (13.73, 14.34) | 13.81 (13.60, 14.18) | 14.11 (13.81, 14.42) | 0.88 (0.74-1.05) | 0.159 | 0.533 | 0.90 (0.75-1.08) | 0.253 | 0.697 | 0.87 (0.73-1.05) | 0.143 | 0.522 | 1.03 (0.90-1.18) | 0.673 | 0.912 |
| **PDLIM5** | 11.81 (10.81, 13.18) | 12.13 (10.82, 13.44) | 11.47 (10.80, 12.87) | 11.66 (10.64, 13.79) | 0.96 (0.52-1.78) | 0.905 | 0.981 | 1.14 (0.59-2.20) | 0.704 | 0.962 | 0.83 (0.43-1.59) | 0.573 | 0.886 | 1.37 (0.85-2.20) | 0.196 | 0.749 |
| **PGF** | 13.73 (13.41, 13.94) | 13.76 (13.45, 13.98) | 13.62 (13.35, 13.90) | 13.58 (13.41, 13.71) | 1.03 (0.93-1.14) | 0.583 | 0.824 | 1.04 (0.93-1.16) | 0.488 | 0.931 | 1.02 (0.92-1.13) | 0.732 | 0.938 | 1.02 (0.94-1.10) | 0.622 | 0.912 |
| **PGK1** | 10.58 (9.99, 11.55) | 10.76 (10.12, 11.64) | 10.51 (9.82, 11.25) | 10.51 (9.91, 11.41) | 1.17 (0.81-1.70) | 0.405 | 0.824 | 1.21 (0.81-1.82) | 0.345 | 0.718 | 1.13 (0.76-1.69) | 0.536 | 0.885 | 1.07 (0.80-1.43) | 0.644 | 0.912 |
| **POSTN** | 14.12 (13.83, 14.39) | 14.19 (13.85, 14.45) | 14.02 (13.81, 14.33) | 14.08 (13.82, 14.39) | 1.05 (0.92-1.20) | 0.468 | 0.824 | 1.10 (0.95-1.27) | 0.202 | 0.687 | 1.01 (0.87-1.17) | 0.898 | 0.975 | 1.09 (0.98-1.21) | 0.112 | 0.736 |
| **PRDX6** | 9.31 (8.65, 10.13) | 9.39 (8.84, 10.13) | 9.22 (8.52, 10.16) | 9.25 (8.50, 9.83) | 1.05 (0.80-1.39) | 0.706 | 0.889 | 1.08 (0.80-1.46) | 0.616 | 0.955 | 1.03 (0.77-1.39) | 0.832 | 0.949 | 1.05 (0.84-1.30) | 0.685 | 0.913 |
| **pSNCA.129** | 12.64 (11.76, 13.52) | 12.69 (11.78, 13.57) | 12.56 (11.71, 13.43) | 12.70 (11.74, 13.35) | 1.00 (0.90-1.11) | 0.965 | 0.988 | 1.03 (0.93-1.16) | 0.539 | 0.955 | 0.97 (0.87-1.08) | 0.52 | 0.885 | 1.07 (0.99-1.16) | 0.084 | 0.654 |
| **PSEN1** | 11.17 (10.92, 11.44) | 11.27 (10.98, 11.49) | 11.08 (10.85, 11.30) | 11.10 (10.88, 11.33) | 0.90 (0.62-1.30) | 0.57 | 0.824 | 0.92 (0.62-1.37) | 0.694 | 0.962 | 0.88 (0.59-1.30) | 0.515 | 0.885 | 1.05 (0.79-1.40) | 0.729 | 0.930 |
| **pTau.181** | 13.32 (12.95, 13.78) | 13.48 (13.09, 13.92) | 13.12 (12.86, 13.45) | 12.97 (12.67, 13.25) | 1.21 (1.02-1.43) | 0.027 | 0.183 | 1.35 (1.13-1.62) | <0.001 | 0.023 | 1.09 (0.92-1.30) | 0.32 | 0.779 | 1.24 (1.09-1.41) | 0.001 | 0.147 |
| **pTau.217** | 11.45 (11.21, 11.72) | 11.48 (11.23, 11.75) | 11.43 (11.20, 11.59) | 11.40 (11.15, 11.62) | 1.07 (0.94-1.21) | 0.288 | 0.685 | 1.10 (0.97-1.26) | 0.147 | 0.553 | 1.04 (0.91-1.18) | 0.57 | 0.886 | 1.06 (0.96-1.17) | 0.218 | 0.749 |
| **pTau.231** | 13.50 (13.25, 13.87) | 13.62 (13.30, 13.92) | 13.38 (13.18, 13.63) | 13.39 (13.12, 13.52) | 1.11 (0.98-1.27) | 0.104 | 0.477 | 1.19 (1.04-1.37) | 0.013 | 0.145 | 1.05 (0.91-1.20) | 0.514 | 0.885 | 1.14 (1.03-1.26) | 0.011 | 0.405 |
| **pTDP43.409** | 11.25 (11.06, 11.56) | 11.26 (11.06, 11.60) | 11.24 (11.07, 11.50) | 11.22 (10.96, 11.44) | 1.07 (0.96-1.18) | 0.216 | 0.627 | 1.09 (0.98-1.22) | 0.114 | 0.472 | 1.04 (0.93-1.16) | 0.447 | 0.859 | 1.05 (0.97-1.14) | 0.25 | 0.772 |
| **PTN** | 9.71 (9.48, 9.92) | 9.72 (9.48, 9.94) | 9.71 (9.48, 9.90) | 9.65 (9.37, 9.82) | 1.09 (0.97-1.21) | 0.148 | 0.533 | 1.12 (0.99-1.26) | 0.07 | 0.378 | 1.06 (0.94-1.19) | 0.353 | 0.825 | 1.06 (0.97-1.15) | 0.213 | 0.749 |
| **REST** | 13.25 (12.92, 13.49) | 13.27 (12.96, 13.51) | 13.21 (12.86, 13.42) | 13.14 (12.92, 13.42) | 0.92 (0.78-1.08) | 0.306 | 0.703 | 0.96 (0.80-1.14) | 0.631 | 0.955 | 0.89 (0.75-1.05) | 0.165 | 0.551 | 1.08 (0.95-1.23) | 0.216 | 0.749 |
| **RUVBL2** | 11.26 (10.77, 12.08) | 11.34 (10.81, 12.05) | 11.14 (10.73, 12.13) | 11.11 (10.44, 11.83) | 1.01 (0.78-1.31) | 0.942 | 0.981 | 1.04 (0.78-1.37) | 0.808 | 0.962 | 0.99 (0.75-1.30) | 0.926 | 0.976 | 1.05 (0.86-1.28) | 0.643 | 0.912 |
| **S100A12** | 13.00 (12.36, 13.69) | 13.08 (12.44, 13.72) | 12.83 (12.27, 13.44) | 13.10 (12.48, 13.40) | 0.95 (0.73-1.25) | 0.713 | 0.889 | 0.99 (0.74-1.33) | 0.95 | 0.969 | 0.92 (0.69-1.22) | 0.549 | 0.885 | 1.08 (0.88-1.34) | 0.464 | 0.906 |
| **S100B** | 13.20 (12.89, 13.48) | 13.22 (12.89, 13.50) | 13.14 (12.86, 13.46) | 13.19 (12.94, 13.53) | 0.95 (0.83-1.10) | 0.519 | 0.824 | 0.94 (0.81-1.10) | 0.437 | 0.846 | 0.97 (0.83-1.12) | 0.662 | 0.938 | 0.97 (0.87-1.09) | 0.631 | 0.912 |
| **SAA1** | 8.92 (7.82, 10.06) | 9.26 (8.09, 10.41) | 8.55 (7.45, 9.72) | 9.33 (8.40, 10.37) | 0.60 (0.37-0.98) | 0.042 | 0.247 | 0.63 (0.37-1.07) | 0.088 | 0.438 | 0.57 (0.34-0.97) | 0.039 | 0.218 | 1.10 (0.75-1.61) | 0.636 | 0.912 |
| **SFRP1** | 10.74 (10.39, 11.91) | 10.87 (10.39, 12.07) | 10.66 (10.35, 11.53) | 10.84 (10.30, 11.62) | 1.00 (0.64-1.56) | 0.988 | 0.988 | 1.00 (0.62-1.62) | 0.995 | 0.995 | 0.99 (0.62-1.60) | 0.983 | 0.983 | 1.00 (0.71-1.42) | 0.983 | 0.995 |
| **SFTPD** | 13.80 (13.37, 14.15) | 13.81 (13.47, 14.17) | 13.74 (13.35, 14.04) | 13.71 (13.41, 14.52) | 0.96 (0.81-1.14) | 0.668 | 0.889 | 1.03 (0.85-1.23) | 0.791 | 0.962 | 0.91 (0.76-1.09) | 0.311 | 0.779 | 1.13 (0.99-1.28) | 0.08 | 0.654 |
| **SLIT2** | 13.73 (13.55, 13.96) | 13.74 (13.54, 14.00) | 13.72 (13.59, 13.85) | 13.69 (13.53, 13.86) | 1.00 (0.91-1.09) | 0.935 | 0.981 | 0.98 (0.89-1.08) | 0.671 | 0.962 | 1.01 (0.92-1.11) | 0.806 | 0.949 | 0.97 (0.91-1.04) | 0.356 | 0.837 |
| **SMOC1** | 11.23 (10.65, 11.76) | 11.28 (10.77, 11.82) | 11.00 (10.49, 11.54) | 11.08 (10.71, 11.59) | 0.92 (0.70-1.22) | 0.574 | 0.824 | 0.98 (0.73-1.32) | 0.913 | 0.969 | 0.87 (0.65-1.17) | 0.366 | 0.825 | 1.12 (0.91-1.39) | 0.278 | 0.784 |
| **SNAP25** | 12.75 (12.61, 12.89) | 12.73 (12.60, 12.87) | 12.78 (12.63, 12.96) | 12.93 (12.79, 13.12) | 0.89 (0.84-0.95) | <0.001 | 0.030 | 0.87 (0.81-0.93) | <0.001 | 0.003 | 0.92 (0.86-0.98) | 0.015 | 0.160 | 0.94 (0.90-0.99) | 0.021 | 0.454 |
| **SNCA** | 12.93 (12.00, 13.88) | 13.01 (12.11, 13.86) | 12.70 (11.79, 13.91) | 12.89 (12.09, 13.77) | 0.95 (0.67-1.35) | 0.771 | 0.928 | 0.99 (0.68-1.44) | 0.945 | 0.969 | 0.92 (0.63-1.33) | 0.648 | 0.938 | 1.08 (0.82-1.41) | 0.597 | 0.912 |
| **SNCB** | 11.68 (11.33, 12.02) | 11.67 (11.31, 12.03) | 11.70 (11.43, 12.01) | 11.54 (11.24, 12.05) | 1.06 (0.90-1.25) | 0.487 | 0.824 | 1.04 (0.87-1.25) | 0.646 | 0.955 | 1.08 (0.90-1.29) | 0.413 | 0.859 | 0.97 (0.85-1.10) | 0.629 | 0.912 |
| **SOD1** | 12.70 (12.05, 13.65) | 12.70 (12.13, 13.65) | 12.62 (11.94, 13.60) | 12.45 (11.64, 13.27) | 1.04 (0.76-1.41) | 0.81 | 0.945 | 1.04 (0.74-1.44) | 0.834 | 0.962 | 1.04 (0.75-1.44) | 0.814 | 0.949 | 1.00 (0.78-1.27) | 0.974 | 0.995 |
| **SQSTM1** | 10.78 (10.47, 11.06) | 10.87 (10.58, 11.15) | 10.63 (10.36, 10.88) | 10.81 (10.34, 11.01) | 0.84 (0.71-0.98) | 0.025 | 0.179 | 0.85 (0.72-1.01) | 0.066 | 0.378 | 0.82 (0.69-0.97) | 0.02 | 0.176 | 1.04 (0.92-1.18) | 0.517 | 0.912 |
| **TAFA5** | 14.21 (13.93, 14.57) | 14.26 (13.96, 14.61) | 14.15 (13.86, 14.54) | 14.55 (14.28, 14.85) | 0.84 (0.73-0.97) | 0.019 | 0.154 | 0.87 (0.75-1.02) | 0.088 | 0.438 | 0.81 (0.70-0.95) | 0.008 | 0.112 | 1.08 (0.96-1.20) | 0.203 | 0.749 |
| **TARDBP** | 12.55 (11.91, 13.07) | 12.62 (11.91, 13.12) | 12.39 (11.93, 12.90) | 12.53 (11.75, 12.79) | 0.94 (0.74-1.18) | 0.585 | 0.824 | 0.96 (0.75-1.24) | 0.761 | 0.962 | 0.92 (0.71-1.17) | 0.487 | 0.885 | 1.05 (0.88-1.26) | 0.597 | 0.912 |
| **TEK** | 13.87 (13.66, 14.04) | 13.89 (13.70, 14.07) | 13.84 (13.62, 13.97) | 13.95 (13.75, 14.04) | 0.95 (0.87-1.04) | 0.283 | 0.685 | 0.97 (0.88-1.07) | 0.517 | 0.949 | 0.94 (0.85-1.03) | 0.185 | 0.589 | 1.03 (0.96-1.11) | 0.359 | 0.837 |
| **TIMP3** | 14.87 (14.17, 15.81) | 15.05 (14.21, 15.94) | 14.74 (14.08, 15.57) | 15.34 (14.47, 16.49) | 0.65 (0.46-0.92) | 0.016 | 0.140 | 0.74 (0.51-1.07) | 0.108 | 0.461 | 0.59 (0.41-0.84) | 0.004 | 0.112 | 1.26 (0.96-1.65) | 0.09 | 0.654 |
| **TNF** | 13.40 (13.21, 13.64) | 13.46 (13.25, 13.69) | 13.31 (13.18, 13.58) | 13.43 (13.17, 13.55) | 0.97 (0.88-1.07) | 0.572 | 0.824 | 0.98 (0.88-1.09) | 0.721 | 0.962 | 0.96 (0.86-1.07) | 0.496 | 0.885 | 1.02 (0.94-1.10) | 0.663 | 0.912 |
| **TREM1** | 13.88 (13.62, 14.12) | 13.93 (13.59, 14.17) | 13.84 (13.63, 14.02) | 13.82 (13.65, 13.99) | 1.04 (0.93-1.15) | 0.506 | 0.824 | 1.07 (0.96-1.19) | 0.248 | 0.697 | 1.01 (0.90-1.12) | 0.898 | 0.975 | 1.06 (0.98-1.15) | 0.154 | 0.749 |
| **TREM2** | 12.10 (11.78, 12.50) | 12.16 (11.83, 12.54) | 11.99 (11.71, 12.30) | 12.41 (11.91, 12.70) | 0.82 (0.70-0.96) | 0.012 | 0.115 | 0.81 (0.69-0.96) | 0.017 | 0.150 | 0.82 (0.70-0.97) | 0.022 | 0.178 | 0.99 (0.87-1.12) | 0.857 | 0.975 |
| **UCHL1** | 9.59 (9.17, 9.91) | 9.64 (9.25, 9.96) | 9.56 (9.03, 9.82) | 9.51 (8.95, 9.92) | 1.08 (0.90-1.28) | 0.402 | 0.824 | 1.12 (0.93-1.36) | 0.222 | 0.687 | 1.04 (0.86-1.25) | 0.707 | 0.938 | 1.09 (0.95-1.24) | 0.238 | 0.757 |
| **VCAM1** | 13.58 (13.41, 13.79) | 13.61 (13.47, 13.84) | 13.52 (13.37, 13.75) | 13.56 (13.41, 13.78) | 1.04 (0.95-1.13) | 0.422 | 0.824 | 1.08 (0.98-1.18) | 0.129 | 0.516 | 1.00 (0.91-1.10) | 0.961 | 0.977 | 1.07 (1.00-1.15) | 0.042 | 0.523 |
| **VEGFA** | 14.58 (14.42, 14.74) | 14.61 (14.46, 14.78) | 14.53 (14.38, 14.70) | 14.64 (14.51, 14.82) | 0.93 (0.86-1.01) | 0.077 | 0.384 | 0.96 (0.88-1.04) | 0.289 | 0.697 | 0.91 (0.84-0.99) | 0.028 | 0.186 | 1.05 (0.99-1.12) | 0.124 | 0.749 |
| **VEGFD** | 12.00 (11.78, 12.25) | 12.02 (11.78, 12.29) | 11.99 (11.78, 12.15) | 12.10 (11.88, 12.29) | 0.97 (0.87-1.07) | 0.531 | 0.824 | 0.99 (0.88-1.10) | 0.832 | 0.962 | 0.95 (0.85-1.06) | 0.355 | 0.825 | 1.04 (0.96-1.13) | 0.331 | 0.826 |
| **VGF** | 12.29 (11.18, 13.19) | 12.37 (11.19, 13.35) | 12.19 (11.15, 12.84) | 12.16 (11.02, 12.85) | 0.90 (0.54-1.50) | 0.676 | 0.889 | 0.91 (0.52-1.58) | 0.725 | 0.962 | 0.89 (0.51-1.54) | 0.672 | 0.938 | 1.02 (0.68-1.52) | 0.926 | 0.992 |
| **VSNL1** | 12.33 (12.07, 12.59) | 12.33 (12.09, 12.59) | 12.34 (12.04, 12.60) | 12.31 (11.99, 12.76) | 0.88 (0.79-0.98) | 0.025 | 0.179 | 0.87 (0.77-0.98) | 0.027 | 0.212 | 0.89 (0.79-1.00) | 0.047 | 0.245 | 0.98 (0.90-1.07) | 0.73 | 0.930 |
| **YWHAZ** | 8.64 (8.08, 9.13) | 8.70 (8.11, 9.16) | 8.51 (7.99, 9.00) | 8.55 (8.24, 9.06) | 0.93 (0.76-1.15) | 0.52 | 0.824 | 0.95 (0.76-1.20) | 0.686 | 0.962 | 0.92 (0.73-1.14) | 0.437 | 0.859 | 1.04 (0.89-1.23) | 0.614 | 0.912 |

*All regressions adjusted for age, sex and body mass index.*

**Supplementary Table 2 . Significant proteomic expression differences in midlife ex-rugby players (overview)**

|  | | | |
| --- | --- | --- | --- |
| **Protein** | ***Fold Change (95% CI)*** | ***P-value*** | ***Adjusted P-value (FDR)*** |
| **Ex-players vs healthy controls** | | | |
| KLK6 | *0.80 (0.72-0.88)* | *<0.001* | *0.001* |
| GFAP | *0.77 (0.67-0.89)* | *<0.001* | *0.020* |
| SNAP25 | *0.89 (0.84-0.95)* | *<0.001* | *0.030* |
| **Ex-forwards vs healthy controls** | | | |
| KLK6 | *0.79 (0.71-0.87)* | *<0.001* | *0.002* |
| GFAP | *0.73 (0.63-0.84)* | *<0.001* | *0.002* |
| SNAP25 | *0.87 (0.81-0.93)* | *<0.001* | *0.003* |
| BACE1 | *0.89 (0.82-0.95)* | *<0.001* | *0.023* |
| pTau-181 | *1.35 (1.13-1.62)* | *<0.001* | *0.023* |
| Aβ38 | *0.77 (0.66-0.90)* | *0.001* | *0.029* |
| **Ex-backs vs healthy controls** | | | |
| KLK6 | *0.81 (0.73-0.90)* | *<0.001* | *0.014* |

*Padj=FDR adjusted P value (<0.05), fold change calculated by exponentiating (2^) the logFC coefficient from the regression. Regression included covariates of age, sex and body mass index.* *GFAP: glial fibrillary acidic protein; KLK6:* kallikrein-6; SNAP25: synaptosomal-associated protein 25; *pTau217: phospho-tau217;*  *Aβ38: amyloid beta 38*

**Supplementary Table 3. Regional brain volumes in ex-players and controls**

|  | **Rugby** N = 197^1^ | **Control** N = 33^1^ |
| --- | --- | --- |
| **Grey matter volume** | 579,090 (546,837, 609,141) | 584,423 (554,972, 615,433) |
| **White matter volume** | 510,497 (475,858, 551,670) | 504,854 (473,899, 562,781) |
| **Hippocampal volume** | 9,082 (8,410, 9,672) | 9,046 (8,531, 9,398) |
| **Ventricular volume** | 19,351 (14,334, 25,296) | 20,891 (16,576, 26,012) |

*^1^* Median (Q1, Q3)

**SUPPLEMENTARY FIGURES**

**Supplementary Figure 1. Biomarker correlations between NULISA and SIMOA platforms**


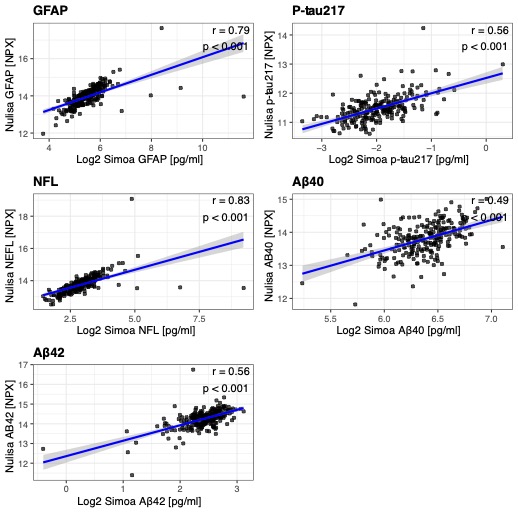


*Correlations of biomarkers on the NULISA platform and corresponding previously-quantified markers using Quanterix SIMOA (log2 transformed). Y axis is normalised protein expression (NPX). Spearman’s correlation coefficient is shown with uncorrected P values. GFAP: glial fibrillary acidic protein; pTau217: phospho-tau_217_; NEFL: neurofilament light; AB40: amyloid beta 40; AB42: amyloid beta 42.*
